# A Green Approach to Natural Dyes in Dye-Sensitized Solar Cells

**DOI:** 10.3390/s23208412

**Published:** 2023-10-12

**Authors:** Nurul Izzati Abdul Shukor, Kah-Yoong Chan, Gregory Soon How Thien, Mian-En Yeoh, Pei-Ling Low, Nisha Kumari Devaraj, Zi-Neng Ng, Boon Kar Yap

**Affiliations:** 1 Centre for Advanced Devices and Systems, Faculty of Engineering, Multimedia University, Persiaran Multimedia, Cyberjaya 63100, Selangor, Malaysianisha@mmu.edu.my (N.K.D.); 2 Intel Corporation, Bayan Lepas 11900, Pulau Pinang, Malaysia; 3 School of Electrical Engineering and Artificial Intelligence, Xiamen University Malaysia, Jalan Sunsuria, Bandar Sunsuria, Sepang 43900, Selangor, Malaysia; 4 Electronic and Communications Department, College of Engineering, Universiti Tenaga Nasional, Kajang 43000, Selangor, Malaysia; 5 Institute of Sustainable Energy, Universiti Tenaga Nasional, Kajang 43000, Selangor, Malaysia; 6 International School of Advanced Materials, South China University of Technology, 381 Wushan Road, Tianhe District, Guangzhou 510640, China

**Keywords:** dye-sensitized solar cells, blueberry, mulberry, anthocyanin, photovoltaics

## Abstract

Solar cells are pivotal in harnessing renewable energy for a greener and more sustainable energy landscape. Nonetheless, eco-friendly materials for solar cells have not been as extensive as conventional counterparts, highlighting a significant area for further investigation in advancing sustainable energy technologies. This study investigated natural dyes from cost-effective and environmentally friendly blueberries and mulberries. These dyes were utilized as alternative sensitizers for dye-sensitized solar cells (DSSCs). Alongside the natural dyes, a green approach was adopted for the DSSC design, encompassing TiO_2_ photoanodes, eco-friendly electrolytes, and green counter-electrodes created from graphite pencils and candle soot. Consequently, the best-optimized dye sensitizer was mulberry, with an output power of 13.79 µW and 0.122 µW for outdoor and indoor environments, respectively. This study underscored the feasibility of integrating DSSCs with sensitizers derived from readily available food ingredients, potentially expanding their applications in educational kits and technology development initiatives.

## 1. Introduction

Synthetic ruthenium (Ru)-based dyes, exemplified by N3 and N719 dyes, have conventionally been the go-to choice for fabricating dye-sensitized solar cells (DSSCs) [1]. These artificial dyes have gained popularity due to their remarkable light absorption properties and ability to facilitate efficient charge transfer through metal–ligand charge transfer transitions [2,3]. While Ru-based dyes have demonstrated the potential to yield highly efficient DSSCs, they come with certain drawbacks, including their elevated cost due to the scarcity of the noble metal ruthenium and the intricate synthesis methods involved [4]. Consequently, there is a growing interest in exploring alternative, more cost-effective, and environmentally friendly options, and this is where natural dyes come into the spotlight [5,6]. Natural dyes from abundant and sustainable sources present an attractive alternative to replace their synthetic counterparts in DSSCs. These dyes are not only cost-effective but also environmentally benign. The literature has previously documented the utilization of natural dyes in DSSCs, with examples including anthocyanin dyes extracted from black rice [7], red cabbages [7,8], and blueberries [9]. Conversely, a comparative analysis between two specific natural dyes (blueberries and mulberries) concerning their efficacy in DSSC design structures and optimizing dye immersion periods remains an unexplored research avenue. Hence, this study aims to bridge this research gap by shedding light on these crucial aspects.

Anthocyanins are inherent pigments that impart red, blue, and purple hues to various fruits, vegetables, and flowers [10]. These substances exhibit water solubility and are primarily located within the vacuoles of plant cells. Anthocyanins have been identified as a viable option for serving as photosensitizers in DSSCs. The photosensitizer, the molecule responsible for light absorption and subsequent electron flow initiation, plays a crucial role in the functioning of DSSCs. Anthocyanins provide several notable advantages compared to synthetic photosensitizers, such as their cost-effectiveness, eco-friendliness, and widespread availability. Nevertheless, anthocyanins do possess certain drawbacks. For instance, anthocyanins are susceptible to degradation when exposed to sunlight and have a limited capacity to absorb light within the visible spectrum efficiently. Notwithstanding these obstacles, anthocyanins exhibit remarkable potential as a viable choice for utilization in DSSCs. Further investigation is required to enhance the subject’s stability and light absorption characteristics. Nevertheless, the utilization of anthocyanins in DSSCs presents noteworthy advantages, which have the potential to foster the advancement of solar cell technology by enhancing efficiency and sustainability.

Solar cells, called photovoltaic (PV) cells, are intricate electronic devices that harness light energy from various sources. These cells operate on the principle of the PV effect, a fascinating interplay of physical and chemical processes [11,12]. When illuminated by light, solar cells undergo dynamic changes in their electrical characteristics, affecting parameters such as current (*I*), voltage (*V*), and resistance (*R*). The history of solar cell development dates to the 1880s, marking the inception of a journey filled with remarkable technological advancements. Solar cells have recently been positioned as a compelling alternative to conventional fossil fuels. This surge in attention is primarily driven by the pressing need for sustainable energy sources in our modern world. Despite the substantial progress, solar cell research remains dynamic and ever-evolving. Researchers and innovators worldwide are intensifying their efforts to push the boundaries of efficiency and effectiveness in solar cell technology. This unwavering commitment to advancement underscores the profound potential of solar cells as a cornerstone of clean energy solutions for the future.

The evolution of technology characterizes the landscape of solar cells into three distinct generations, each catering to diverse requirements and functions. The initial generation saw the deployment of traditional wafer-based cells, marking the inception of solar cell technology. Following this, the second generation emerged, showcasing the advent of thin-film technologies. This group encompasses materials like copper indium gallium selenide (CIGS) [13,14], cadmium telluride (CdTe) [15], gallium arsenide (GaAs) [16,17], and amorphous silicon (a-Si: H), among others, which have been widely explored [18]. In contemporary solar cell research, first- and second-generation cells continue to draw significant attention due to their prevalence in commercial markets. Nevertheless, the quest for alternative solar cell types gained momentum as PVs approached theoretical performance limits [19,20]. This search has given rise to the development of third-generation solar cells, representing a novel and promising frontier in PV technology. These third-generation cells hold the potential to surpass the existing technologies, potentially revolutionizing the field of solar energy.

DSSCs represent an economic class of solar cells categorized within the third generation of solar cell technologies. These DSSCs can be attributed to the pioneering work of Michael Gratzel in the 1970s. Gratzel’s innovative approach involved the utilization of oxide semiconductors, dye-based sensitizers, and an electrolyte in these cells. In a notable development in 1991, Michael Gratzel and Brian O’Regan reached a remarkable milestone by enhancing the efficiency of DSSCs by 7.12% [21]. This breakthrough propelled the further exploration of DSSCs due to their immense potential for future advancements. One of the distinguishing features of DSSCs is their cost-effectiveness, making them a highly attractive option in solar cell technology. The manufacturing costs associated with DSSCs are relatively modest, offering the prospect of affordable large-scale production [22]. Unlike other types of solar cells that rely on specific light intensity ranges, DSSCs exhibit a unique advantage—they tend to perform optimally even under overcast skies and in low light conditions, with minimal impact on their efficiency. Adapting to varying light conditions enhances their practicality and appeal in real-world applications [23].

A DSSC is a PV device that employs a dye molecule to capture solar radiation and transform it into electrical energy [24]. The fundamental configuration of a DSSC comprises a transparent conducting oxide (TCO) electrode, a dye-sensitized semiconductor layer, an electrolyte, and a counter-electrode. The TCO electrode facilitates light transmission and propagation towards the dye-sensitized semiconductor. The dye-sensitized semiconductor is a permeable layer composed of a semiconductor substance, specifically titanium dioxide (TiO_2_), enveloped by a dye molecule [1,25]. When the incident sunlight interacts with the dye molecule, it undergoes absorption, leading to the excitation of one electron within the molecule. Subsequently, the electron in an excited state is transferred to the semiconductor material, enabling its movement along an external circuit and facilitating the production of electrical energy. The electrolyte solution facilitates the transfer of electrons between the dye molecule and the semiconductor. Finally, the counter-electrode, or the auxiliary electrode, serves the purpose of electron collection from the semiconductor material.

This study was dedicated to creating DSSCs using common culinary ingredients, making the process accessible and adaptable in various settings. The study encompassed the entire DSSC production process, a thorough characterization of DSSCs under diverse conditions, and an exploration of the factors influencing the performance of these solar cells. This study also involved the meticulous design of an engineering kit centered around DSSCs. The PV properties of DSSCs were assessed utilizing natural-based components. Its environmentally friendly approach to solar cell fabrication sets this engineering kit apart from conventional DSSCs. The kit incorporated dyes derived from blueberries and mulberries, a pharmaceutical electrolyte solution, TiO_2_ photoanodes, and green counter-electrodes to establish a complete circuit. The performance of the green solar cell kit was thoroughly analyzed by varying parameters such as the type of photosensitizer, immersion time for dye extraction, and the composition of the counter-electrodes within the circuit.

## 2. Materials and Methods

### 2.1. Preparation of Natural Dyes

Natural dyes are derived from plants, animals, or minerals. They are a sustainable and environmentally friendly alternative to synthetic dyes. In this study, two types of natural dyes were prepared: blueberry dye and mulberry dye. Initially, fresh blueberries were procured from a local supermarket. The blueberries were carefully placed inside a clean plastic bag and thoroughly crushed to extract their dyes. Subsequently, 5 ml of deionized water was added to the crushed blueberries to dilute the natural dye. Any excess blueberry remnants were filtered out to achieve a homogeneous dye solution. Meanwhile, the process for obtaining mulberry-based dye was replicated following the same procedure outlined above. The resulting dye solutions were dark purple in color. The blueberry dye was slightly more vibrant than the mulberry dye.

### 2.2. Preparation of DSSCs

**Figure 1:**
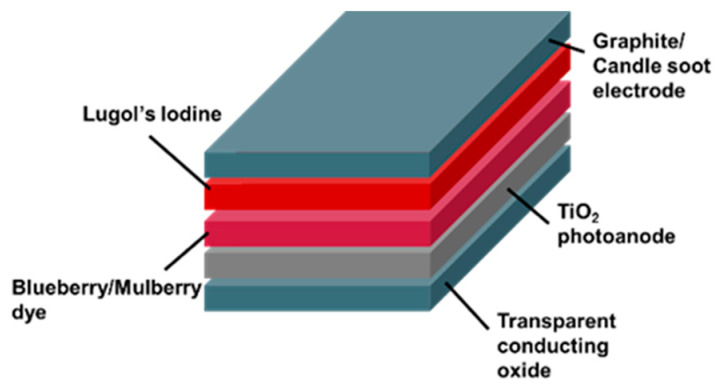
The design structure of the DSSC device is based on a green approach.

The design structure is constructed following the architecture in Figure 1. Initially, fluorine-doped tin oxide (FTO) substrates underwent ultrasonic cleaning with acetone and isopropyl alcohol for 5 min each. Subsequently, TiO_2_ photoanodes were prepared using commercial TiO_2_ paste (Ti-Nanoxide D, Solaronix, Foothill Ranch, CA, USA) and applied to the FTO substrates using a glass rod using the doctor blade method. The TiO_2_ photoanodes were then sintered on a hot plate at 450 °C for 45 min and cooled to room temperature. Table 1 tabulates four different types of DSSCs. The first DSSC device (Exp I) utilized blueberry extract as the dye and graphite as the counter-electrode, with a 5 min dye immersion time. The second DSSC device (Exp II) replaced blueberry with mulberry extract, keeping other parameters constant. The third DSSC device (Exp III) replicated Exp II but extended the dye immersion time to 12 h. The fourth DSSC device (Exp IV) mirrored Exp I but replaced graphite with candle soot as the counter-electrode. Specific parameters were standardized to ensure maximum output and comparable results across the experiment, such as dye soaking time in the electrolyte solution, dye extraction from five pieces of blueberry, and 5 ml of water solvent.

Following the dye extraction, binder clips were employed to assemble both electrodes and create a secure electrode bond. Subsequently, the electrolyte solution was injected between the electrodes to enhance conductivity, effectively completing the assembly of the DSSCs. Data collection commenced by monitoring the performance and degradation of the DSSCs over fixed time intervals. The DSSCs underwent testing in both real-life outdoor and indoor conditions. For the outdoor evaluation, the DSSC was positioned directly under natural sunlight, specifically during a period with low cloud cover, at 2 p.m. (GMT +8), replicating real-life outdoor environmental conditions. In contrast, for the indoor assessment, the DSSCs were placed beneath fluorescent lighting at a height of 2.15 m from the ground, and the tests were conducted at 9 p.m. to simulate indoor environmental conditions.

### 2.3. Measurement of DSSC Performance

The evaluation of DSSC performance was conducted using a digital multimeter. This process involved connecting the positive probe of the multimeter to the working electrode and the negative probe to the counter-electrode. Subsequently, the voltage and current produced by each DSSC were measured and analyzed. This study focused on assessing the solar cell’s performance with various parameters.

## 3. Results and Discussion

### 3.1. Influence of Photosensitizers

This study selected blueberries and mulberries, which are readily accessible and frequently consumed in many geographical areas. This characteristic enabled the acquisition of fresh samples for experimental purposes. Furthermore, these fruits have gained recognition due to their substantial concentration of anthocyanins, primarily found in their skin or outer layers. The impact of natural photosensitizers, specifically blueberry and mulberry-based dyes, was thoroughly investigated. Table 2 compares the PV responses of DSSCs using these dyes. Notably, the outdoor testing environment consistently yielded higher output power than the indoor environment, indicating the influence of light source intensity. Blueberry-based DSSCs exhibited a remarkable 90-fold increase in power output (7.45 µW) in outdoor sunlight compared to indoor conditions (0.08024 µW).

Similarly, mulberry-based DSSCs displayed an even more substantial difference, with outdoor power output (13.79 µW) being 113-fold higher than indoor conditions (0.122 µW). This significant disparity was attributed to the variation in light intensity between the two environments, a factor known to impact DSSC PV performance [26,27]. The observed phenomenon was explained by the impact of light intensity on the diffusion rate within the solar cell. Higher photon emission in brighter light conditions led to an accelerated diffusion rate. Consequently, a higher current was generated and shifted towards higher open-circuit voltage (V_oc_) values. Ultimately, these elevated V_oc_ values contributed to the larger output power observed in higher light-intensity conditions [28].

**Figure 2:**
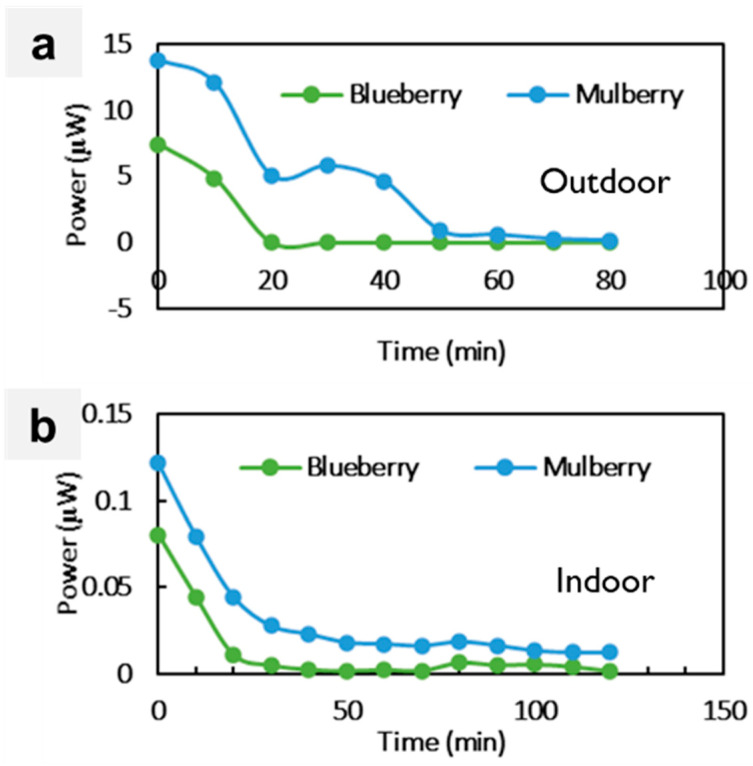
Performances of output power for blueberry and mulberry dyes in (**a**) outdoor and (**b**) indoor conditions.

**Figure 3:**
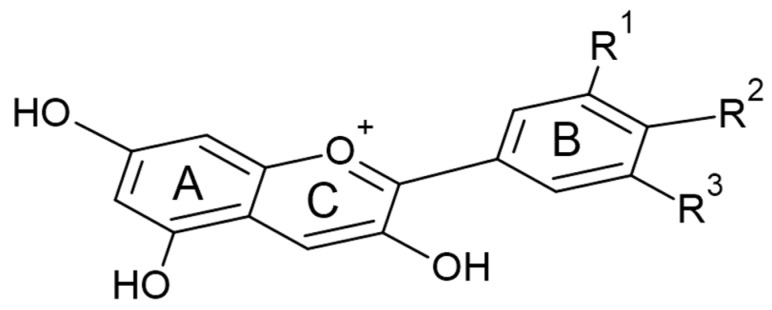
The general chemical structure of anthocyanin in blueberries and mulberries.

Figure 2 clearly illustrates that the performance of mulberry dye DSSCs surpasses that of blueberry dye DSSCs. This superiority was attributed to the higher concentration of anthocyanin pigment found in mulberries, which was evident from the depth of the color dye adsorbed on the TiO_2_ substrate (see Figure 3). Anthocyanins are a class of polyphenolic compounds responsible for the red pigmentation observed in various plant tissues [29]. The chemical structure of anthocyanins is crucial to their ability to absorb sunlight and transmit energy to electrons in a DSSC’s semiconductor layer. Anthocyanidins are the portion of the molecule that absorbs light, whereas sugar molecules stabilize anthocyanins and make them soluble in the electrolyte solution [30].

In addition to anthocyanins, blueberry and mulberry dyes encompass various chemicals. These compounds include flavonoids, carotenoids, organic acids, and polyphenols, typically present in these fruits [31,32]. These compounds have the potential to make a substantial contribution to the overall intensity of color and light-absorption characteristics shown by the dyes. For example, flavonoids have the potential to contribute to increased pigmentation, thus augmenting the overall coloration. In contrast, carotenoids can effectively capture light across a range of wavelengths, expanding the scope of light absorption within the dye-sensitized layer. The presence of organic acids inside the dyes can also impact the pH of the dye solution, potentially influencing the performance of DSSCs by altering the chemical reactions. Meanwhile, the polyphenols included in the dyes can interact with the dye-sensitized layer and the semiconductor, potentially influencing the electron transfer mechanisms inside DSSCs [33]. Considering these substances’ existence and possible impacts offers a more holistic viewpoint on the complex elements that contribute to the intensity of color and performance of DSSCs in systems based on blueberries and mulberries.

The number of hydroxyl groups on the anthocyanidin influences the absorption wavelength. Anthocyanins with a greater number of hydroxyl groups absorb blue light, whereas those with a smaller number of hydroxyl groups absorb red light [34]. Therefore, anthocyanins can give plants a wide spectrum of colors, including blue, red, and purple. The sugar molecules also affect the anthocyanin absorption spectrum. Sugars with more hydroxyl groups cause anthocyanins to absorb more blue light, whereas sugars with fewer hydroxyl groups cause them to absorb more red light [35]. Anthocyanins’ stability in DSSCs also depends on their chemical structure. Stabler anthocyanins are less likely to degrade under the severe conditions of a DSSC. For use in DSSCs, selecting anthocyanins with an optimal balance of absorption and stability properties is crucial.

Anthocyanins can be used as sensitizers in DSSCs, which absorb solar energy and transmit it to the semiconductor layer’s electrons. The excited electrons travel through the semiconductor to the metal electrode, producing an electrical current. Considering that anthocyanins are non-toxic, biodegradable, and abundant, they are a promising alternative to traditional sensitizers in DSSCs. Additionally, they are affordable to extract from plants. In the case of mulberries, their dye presented a deeper red-purplish hue compared to that of blueberries [36]. Consequently, mulberry dye contained a greater overall quantity of anthocyanin. This heightened concentration of anthocyanin in mulberry dye had a significant impact on solar cell efficiency. The absorbing characteristics of the dye were improved, leading to more efficient light absorption. As a result, the greater amount of anthocyanin in mulberry dye contributed to the improved performance of the associated DSSCs.

### 3.2. Influence of Immersion Time

**Figure 4:**
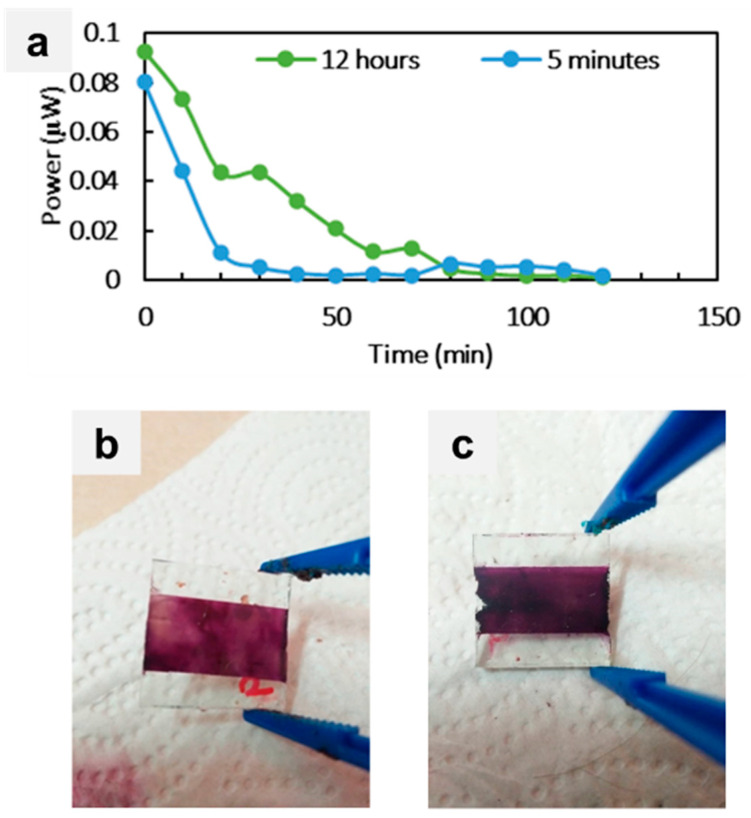
(**a**) Output power performance concerning the different soaking times of dye extraction. Photographs of TiO_2_ photoanodes immersed in the blueberry dye for (**b**) 5 min and (**c**) 12 h.

The influence of the immersion time of TiO_2_ photoanodes in natural dye was investigated (see Table 3). The natural blueberry dye sensitizer was selected for this study, and the impact of varying immersion hours on DSSC performance was assessed indoors. Figure 4a visually compares DSSCs fabricated using different immersion times, specifically shorter soaking times of 5 mins and longer soaking times of 12 h, for the dye extraction process. The outcomes of this investigation demonstrated that the DSSCs with extended immersion times (12 h) displayed complete coverage of dye deposition within the DSSC design structure (see Figure 4b,c). Consequently, a slight increase in power values (from 0.08024 µW to 0.09284 µW) was observed when transitioning from 5 mins to 12 h of soaking time. This increase was attributed to the improved coverage of the dye layer, facilitating enhanced charge transfer from the conduction band of the TiO_2_ surface to the electrolyte [37]. The mechanism behind this improvement in charge transfer was the higher number of electrons transferred within the photoelectrodes. As a result, more electrons in DSSCs were transferred to the external circuit, increasing the overall output power (see Figure 4a).

The optimization of the natural dye sensitizer revealed distinct PV response behaviors with varying immersion hours. Notably, the PV response of the DSSCs improved as the immersion time increased. This outcome suggested that longer immersion times contributed to enhanced DSSC performance. Nonetheless, an intriguing phenomenon occurred when the immersion time surpassed the optimum duration. At this point, the PV response began to degrade. This degradation was attributed to the increased instability of the dye sensitizer under prolonged immersion, suggesting an optimal point beyond which prolonged soaking was detrimental to DSSC performance [38]. The degradation and loss of activity of anthocyanin molecules occurred due to their instability when subjected to extended immersion. Anthocyanins are known to be unstable and can degrade under prolonged exposure to light, heat, and oxygen. In the context of DSSCs, the prolonged immersion of anthocyanin molecules in the electrolyte solution can cause them to degrade and lose their ability to absorb light and transfer electrons [28,39,40]. This outcome can lead to a decrease in the PV response of the DSSCs. Thus, the results of this study suggested that a soaking time of 12 h was a good starting point for achieving optimal performance. Further studies are needed to identify the factors that affect the stability of anthocyanin molecules in DSSCs and to develop methods to improve their stability.

### 3.3. Influence of Counter-Electrodes

**Figure 5:**
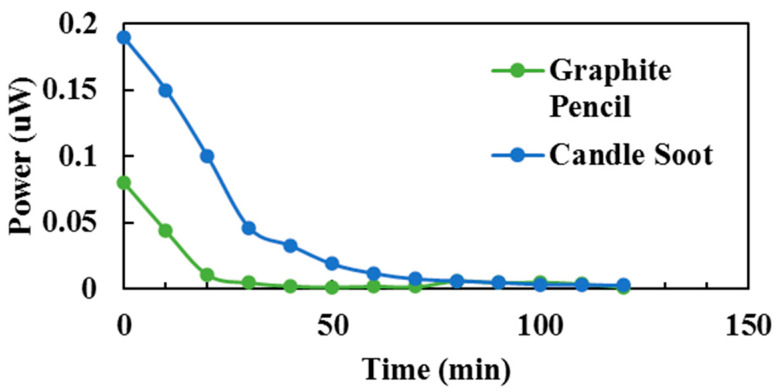
Performance of output power with relation to different types of counter-electrode materials.

The impact of different counter-electrodes was a focal point in this subsection (Section 3.3). Table 4 outlines the PV responses of DSSCs with various counter-electrodes, utilizing graphite pencil and candle soot. These DSSCs were sensitized with blueberry dye and tested in an indoor environment. Furthermore, Figure 5 graphically illustrates the performance of DSSCs fabricated with candle soot and graphite pencil counter-electrodes. Notably, the candle soot-based sample demonstrated a twofold increase in PV response, generating an output power of 0.18954 µW. The efficiency of a solar cell was intricately linked to the transfer of electrolytic charge. In this context, the electrolytic charge from the redox couple I_−_/I_3−_ electrolyte effectively infiltrated and traversed the pores of the candle flame carbon atoms [41]. Consequently, this process facilitated a higher rate of electrolytic charge transfer, ultimately resulting in the heightened efficiency of the DSSCs [42].

Furthermore, it is important to note that the roughness of the carbon electrode surface significantly impacted the solar cell’s performance. Candle-soot-derived carbon exhibited a higher surface resistivity and superior adhesion compared to the graphite layer applied using graphite pencils. These characteristics contributed to a notably improved PV response in the candle soot-based samples [42].

### 3.4. DSSC as an Engineering Teaching Kit

**Figure 6:**
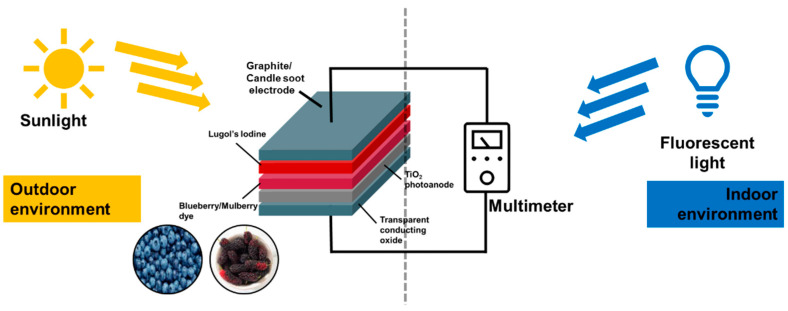
Schematic diagram of the natural dye-based DSSCs measured in indoor and outdoor environments as an engineering teaching kit.

This study developed a straightforward engineering teaching kit based on the DSSC design structure (see Figure 6). The kit comprised TCOs, such as fluorine-doped tin oxide (FTO); TiO_2_; natural dyes extracted from common food ingredients, such as blueberries and mulberries; Lugol’s iodine as the liquid electrolyte; and carbon-based electrodes, such as graphite pencils and candle soot. Using natural dyes as sensitizers in DSSCs is a promising approach as it is eco-friendly and sustainable. The dyes can be extracted from various food and plant materials, and they are non-toxic and biodegradable. Carbon-based electrodes are also sustainable, as they can be made from recycled materials. The engineering kit developed in this study is simple to use and affordable. It can be used to assess the performance of DSSCs in terms of their open-circuit voltage (V*_oc_*), short-circuit current (I*_sc_*), fill factor (FF), and efficiency (*η*). The evaluation process is facilitated using a digital multimeter.

The development of this engineering kit provides a way to educate students about the principles of DSSCs and their potential applications. It also provides a platform for students to conduct their experiments and research into the development of more efficient and sustainable DSSCs. Additional details about the engineering kit can be used to teach students about the following concepts:

1. The basics of solar cells: Understand the basic principles of solar cells, including how they convert light energy into electrical energy; Be able to distinguish between the different types of solar cells, such as silicon solar cells and DSSCs; Understand the factors that affect the efficiency of solar cells.
2. The working principle of DSSCs: Understand the working principle of DSSCs, including how the dye molecules absorb sunlight and transfer their energy to electrons in the TiO_2_ semiconductor; Be able to explain the role of the different components of a DSSC; Understand the factors that affect the efficiency of DSSCs.
3. The different components of DSSCs: Identify the various components of a DSSC; Understand the function of each element; Explain how the other parts interact.
4. The impact of different parameters on the performance of DSSCs: Understand the impact of the other parameters on the performance of DSSCs, such as the type of dye used, the thickness of the TiO_2_ layer, and the electrolyte solution; Be able to design and conduct experiments to investigate the impact of different parameters on the performance of DSSCs.

The kit can also be used to conduct experiments on the following topics:

1. **How to extract natural dyes from food and plant materials:** Understand the principles of dye extraction; Be able to select the appropriate solvent for extracting natural dyes from food and plant materials; Be able to carry out the dye extraction process.
2. **How to prepare DSSCs using natural dyes:** Understand the steps involved in preparing DSSCs using natural dyes; Be able to prepare DSSCs using natural dyes; Be able to evaluate the performance of DSSCs designed using natural dyes.
3. **How to optimize the performance of DSSCs:** Understand the factors that affect the performance of DSSCs; Be able to design and conduct experiments to maximize the performance of DSSCs; Be able to identify the limitations of DSSCs and suggest ways to improve their performance.

Based on the DSSC design structure, this study devised a straightforward engineering teaching kit. Students can learn about the fundamentals of DSSCs and their prospective applications with this kit. It can also be used for experiments involving the extraction of natural dyes from food and plant materials, the preparation of DSSCs utilizing natural dyes, and the optimization of the performance of DSSCs. The kit’s affordability and ease of use make it a valuable resource for teaching and research in DSSCs.

## 4. Conclusions

This study successfully integrated natural dyes derived from common food ingredients, specifically mulberries and blueberries, into DSSCs. Solar cell optimization was conducted through variations in natural dye types, prolonged dye extraction times, and different counter-electrode materials. The PV performance of these fabricated cells was meticulously evaluated, focusing on output power and efficiency. Mulberry-based dye emerged as the best sensitizer, producing an output power of 13.79 µW outdoors and 0.122 µW indoors. Extending the soaking time during dye extraction yielded 0.09284 µW output power; 12 h of immersion was a good starting point for achieving optimal performance. Meanwhile, candle flame carbon employed as the counter-electrode material resulted in an impressive 0.18954 µW output power. Despite this study’s relatively moderate output power, it highlighted the potential of using food ingredient-based sensitizers in DSSCs. This approach could be incorporated into a straightforward engineering kit for educational and technological purposes.

## Figures and Tables

**Table 1:** Summary of DSSCs with their corresponding manipulated variables.

Layer	Exp I	Exp II	Exp III	Exp IV
Anode	TiO_2_	TiO_2_	TiO_2_	TiO_2_
Dye	Blueberry	Mulberry	Blueberry	Blueberry
Electrolyte	Lugol’s iodine	Lugol’s iodine	Lugol’s iodine	Lugol’s iodine
Cathode	Graphite pencil	Graphite pencil	Graphite pencil	Candle soot
Immersion time (min)	5	5	720	5

**Table 2:** Summary of DSSC parameters of blueberry and mulberry natural dyes in outdoor and indoor environments.

Natural Dye	Environment	Voltage, *V_oc_* (mV)	Current, *I_sc_* (µA)	Power, P (µW)
Blueberry	Outdoor	105.8	70.4	7.45
Blueberry	Indoor	23.6	3.4	0.08024
Mulberry	Outdoor	137.5	100.3	13.79
Mulberry	Indoor	29.8	4.1	0.122

**Table 3:** Summary of the PV response of DSSCs from different soaking times.

Exp	Voltage, *V_oc_* (mV)	Current, *I_sc_* (µA)	Power, P (µW)
I	23.6	3.4	0.08024
III	21.1	4.4	0.09284

**Table 4:** Summary of PV response of DSSCs fabricated using graphite and candle soot counter-electrodes.

Exp	Voltage, *V_oc_* (mV)	Current, *I_sc_* (µA)	Power, P (µW)
I	23.6	3.4	0.08024
IV	35.1	5.4	0.18954

## Data Availability

The data presented in this study are available on request from the corresponding author. The data are not publicly available due to privacy reasons.
